# Talpid3-Binding Centrosomal Protein Cep120 Is Required for Centriole Duplication and Proliferation of Cerebellar Granule Neuron Progenitors

**DOI:** 10.1371/journal.pone.0107943

**Published:** 2014-09-24

**Authors:** Chuanqing Wu, Mei Yang, Juan Li, Chengbing Wang, Ting Cao, Kaixiong Tao, Baolin Wang

**Affiliations:** 1 Department of General Surgery, Union Hospital, Tongji Medical College, Huazhong University of Science and Technology, Wuhan, Hubei, China; 2 Department of Genetic Medicine, Weill Medical College of Cornell University, New York, New York, United States of America; 3 Department of Cell Biology and Development, Weill Medical College of Cornell University, New York, New York, United States of America; 4 Department of Human Anatomy, Institute of Neuroscience, Chongqing Medical University, Chongqing, China; 5 Institute of Developmental Immunology, College of Life Science, Shandong University, Jinan, Shandong, China; 6 Institute of Life Science, Nanjing University, Nanjing, Jiangsu, China; Institute of Molecular and Cell Biology, Singapore

## Abstract

Granule neuron progenitors (GNPs) are the most abundant neuronal type in the cerebellum. GNP proliferation and thus cerebellar development require Sonic hedgehog (Shh) secreted from Purkinje cells. Shh signaling occurs in primary cilia originating from the mother centriole. Centrioles replicate only once during a typical cell cycle and are responsible for mitotic spindle assembly and organization. Recent studies have linked cilia function to cerebellar morphogenesis, but the role of centriole duplication in cerebellar development is not known. Here we show that centrosomal protein Cep120 is asymmetrically localized to the daughter centriole through its interaction with Talpid3 (Ta3), another centrosomal protein. Cep120 null mutant mice die in early gestation with abnormal heart looping. Inactivation of Cep120 in the central nervous system leads to both hydrocephalus, due to the loss of cilia on ependymal cells, and severe cerebellar hypoplasia, due to the failed proliferation of GNPs. The mutant GNPs lack Hedgehog pathway activity. Cell biological studies show that the loss of Cep120 results in failed centriole duplication and consequently ciliogenesis, which together underlie Cep120 mutant cerebellar hypoplasia. Thus, our study for the first time links a centrosomal protein necessary for centriole duplication to cerebellar morphogenesis.

## Introduction

The cerebellum controls motor movement coordination, balance, equilibrium, and muscle tone. Granule neurons are the most abundant neurons in the cerebellum and in fact the entire brain. Cerebellar development is a complex process requiring coordinated regulation of progenitor proliferation, neuronal differentiation and migration [1], [2]. Granule neuron progenitors (GNPs) originate in the rhombic lip (RL), a region of the hindbrain bordering the fourth ventricle. In the moue, the GNPs leave the RL at around embryonic day 13 (E13) and migrate rostrally over the surface of the cerebellar anlage to form the external germinal layer (EGL). During the first two weeks after birth, the GNPs in the EGL undergo significant proliferation to generate the large pool of GNPs required for producing granule neurons. They then exit cell cycle and migrate internally into the cortex of the cerebellum to establish the inner granule layer (IGL) under the Purkinje cell monolayer (PCL) [3]. During the same extended period, the cerebellum becomes foliated, significantly increasing its size and surface area. Defects in any of these stages of cerebellar development result in cerebellar hypoplasia in humans and mice.

Sonic Hedgehog (Shh) is a secreted molecule that plays important roles in embryonic development. In the developing cerebellum, Shh is expressed in Purkinje cells and controls GNP proliferation [4]–[7] in part by upregulating the protooncogene N-myc [8]. Hedgehog (Hh) signaling occurs in the primary cilium, a microtubule-based organelle that protrudes from the cell surface of most vertebrate cells [9], including both Purkinje cells and GNPs in the EGL [10], [11]. Defects in ciliogenesis are associated with human congenital cerebellar malformations [12]. Mutations in several ciliary genes in the mouse have also been shown to cause significantly reduced GNP proliferation and cerebellar hypoplasia [13], [14].

Primary cilia originate from the basal body, a specialized mother centriole, during the G0 phase of cell cycle [15]. Centrioles are cylindrical microtubule-based structures, and a typical cell contains two centrioles, the core components of a centrosome. Before cells enter the cell cycle, primary cilia have to be disassembled, and the two centrioles replicate, a process that is strictly controlled to occur only once per cell cycle for a typical cell. As a result, the two centrioles are distinct in age and maturity as well as function. The younger of the two centrioles is known as daughter centriole, and the older one is called mother centriole. During the G0 phase, the mother centriole matures and becomes the basal body of the primary cilium [16]. Thus, centriole duplication and ciliogenesis are two closely related processes. The list of known centrosomal proteins that have been found to be essential for ciliogenesis keeps increasing, but only a small number of them are also required for centriole duplication [17]. Whether these centrosomal proteins regulate cerebellar development is still unknown.

In the present study, we identify the centrosomal protein Cep120 as an interacting protein of Talpid3 (Ta3), another centrosomal protein that is required for ciliogenesis and Hh signaling [18], [19]. Cep120 is asymmetrically localized to the daughter centriole [20], while Ta3 is predominantly found in the mother centriole. The asymmetrical localization of Cep120 is dependent on Ta3. *Cep120* null mutant mice die at an early gestation stage with defect in heart looping. Most *Cep120* mutant cells either lack centrioles or only have one, confirming a recent study showing that Cep120 is required for centriole duplication [20]. Inactivation of Cep120 in the central nervous system results in severe hydrocephalus and cerebellar hypoplasia, subsequently causing lethality. In the Cep120 mutant, cilia are also absent on ependymal cells and cerebellar GNPs (CGNPs) and centriole duplication fails to occur in CGNPs. As a result, the CGNPs lack Hh pathway activity, thus proliferating. The mutant cerebellum also never becomes foliated. These phenotypes are more severe than those of the known cilia mutants [13], [14]. Therefore, *Cep120* mutant cerebellar phenotypes are the result of both failed ciliogenesis and centriole duplication. This study shows for the first time that a centrosomal protein necessary for centriole duplication is also important in cerebellar morphogenesis.

## Materials and Methods

Institutional Animal Care and Use Committee at Weill Cornell Medical College approved this research including the use of mice and mouse embryonic fibroblasts. The protocol number is 2010-0101. All relevant data are within the paper and fully available without restriction.

### Mouse strains and the generation of the *Cep120^-^* and *Cep120^f^* mutant allele

A BAC clone containing mouse Cep120 genomic DNA sequences was purchased from the BACPAC Resources Center (Oakland, CA) and used to create a Cep120 conditional targeting construct. The construct was engineered by inserting a neomycin cassette flanked by two Frt sites, as well as one loxP site upstream and another downstream the fourth exon of the *Cep120* gene. The linearized construct was electroporated into W4 ES cells, and targeted ES cell clones were selected in the medium with G418 (150 µg/ml) and identified by digestion of genomic DNA with BglII, followed by a Southern blot analysis of ES cell DNA using a 3′-probe. Two Cep120 targeted ES cell clones were injected into C57BL/6 blastocysts to generate chimeric founders, which were then bred with C57BL/6 to establish F1 heterozygotes. The *Cep120^-^* allele was created by breeding the F1 mice with *actin-Cre* mice, whereas the *Cep120^f^* allele was generated by crossing the F1 mice with *actin-Flpe* mice to remove the neomycin gene. The mice were maintained in a 129/SVE and C57BL/6 mixed background. PCR (polymerase chain reaction) analysis was used for routine genotyping with the following primers: BW946F, 5′-ATCACTGTGGAGCCTTGGGCA-3′, and BW946R, 5′-TGTTACTCAGCAGCTGGTACC-3′, for the wild type allele, which produced a 220 bp fragment; BW1241F, 5′- CCTCTGCCTCCTTAGTGGATC-3′, and BW946R, for the *Cep120^f^* allele, which produced a 280 bp fragment; BW294, 5′-ATTGGGAAGACAATAGCAGGCA-3′ and BW947, 5′-AGACCAGCCTCAGTTACACCG-3′ for *Cep120^-^* allele, which produced a 230 bp fragment. *Ta3* knockout mice were created by the deletion of the first two exons of the *Ta3* gene using the standard targeted gene knockout approach, and the detail of the strategy will be described elsewhere. *Nestin-Cre* or *nes-Cre* mice were obtained from Dr. Tao Sun and originally purchased from Jackson Laboratories. *Actin-Flpe* and *actin-Cre* mice were obtained from Dr. Licia Selleri and originally purchased from Jackson Laboratories. Since DNA recombination from maternally inherited *nes-Cre* allele is less efficient than that from paternally inherited allele [21], for all crosses involving *nes-Cre*, only the males carrying the transgene were used for crosses. All animal care was done in accordance with the guidelines of the National Institutes of Health.

### Cell Lines and Cell Culture

Wild type, *Cep120^-/-^*, and Ta3 primary mouse embryonic fibroblasts (pMEFs) were prepared from E9.5 and E10.5 mouse embryos, respectively. The procedure was approved by Institutional Animal Care and Use Committee at Weill Medical College. The pMEFs and HEK293 cells were cultured in DMEM supplemented with heat-inactivated 10% FBS, penicillin, and streptomycin. The calcium phosphate precipitation method was used to transfect HEK293 cells as described [22].

### cDNA Constructs and yeast two-hybrid screen

Mouse full-length Cep120 and Ta3 cDNAs were purchased from Thermo Scientific Inc. Cep120 cDNA was cloned into the pCMV-3xFlag vector (Sigma) by restriction enzyme digestion and/or PCR, and full-length Ta3 cDNA and its deletion and truncation mutant constructs were generated using a CMV-based pRK vector. The yeast two-hybrid screen was performed using pNLex-Ta3CT (901–1505 aa) as bait and a cDNA library as described [23]. All constructs were verified by DNA sequencing.

### Tissue immunohistochemistry, cresyl violet staining, BrdU incorporation and TUNEL assays

For BrdU incorporation assay, mice were subcutaneously injected with BrdU (50 µg/g body weight in PBS) 2 h before sacrifice. Brains were dissected from P1, P7, and P14 wild type and *Cep120^f/-^*; *nes-Cre* mice and fixed in 4% paraformaldehyde (PFA)/PBS overnight at 4°C. After equilibration in 30% sucrose/PBS overnight at 4°C, the brains were embedded in OCT and sagittally cryosectioned (10 µm). The tissue sections were incubated with 2N HCl/1%Triton X-100 for 30 min to remove histones, washed with PBS for 3 times, and stained with anti-BrdU antibody. For TUNEL assay, the brain sections from mice without administration with BrdU were incubated with proteinase K (10 ng/ml in PBS) at room temperature for 5 min. After refixing with 4%PFA/PBS for 15 min, the sections were incubated with DNA terminal transferase (TdT, NEB) and BrdU in TdT buffer at 37°C for 1 h. After washing with PBS, the tissue sections were stained with anti-BrdU antibody. For immunofluorescence, the brain sections were stained with the indicated primary antibodies as described [22]. Cresyl violet staining of frozen brain sections was performed by incubating the tissue sections with 0.5% cresyl violet for 1–5 min at room temperature followed by wash with water and dehydration by graded ethanol for 2 min each.

### Cell immunofluorescence and microscopy

Cell immunofluorescence and microscopy, Immunoprecipitation, and immunoblotting were performed as described [22].

### Antibodies

Custom Cep120, Ta3, Odf2, Cep164 antibodies were generated by Covance, Inc. Rabbits were immunized with insoluble His-tagged mouse Cep120 (660-E aa), Ta3 (1–430 aa), Odf2 (full-length), Cep164 (1–298 aa) protein fragments purified from bacteria. All these antibodies were used at a 1∶1000 dilution for both immunoblotting and immunostaining. Other antibodies used for immunofluorescence and Western blotting included Flag M2 mAb (1∶1000), acetylated tubulin (1∶2000), γ-tubulin (1∶2000) (Sigma), Arl13b (1∶1500) [24], Calb1 (1∶200, cat #13176, Cell signaling), Pax6 (1∶50), and BrdU (1∶200) (DSHB, University of Iowa). Secondary antibodies Dylight 488-conjugated donkey anti rabbit IgG and Cy3-conjugated donkey anti mouse IgG were purchased from Jackson Immunoresearch, Inc.

## Results

### Ta3 interacts with and regulates asymmetrical localization of Cep120 to the daughter centrioles

**Figure 1:**
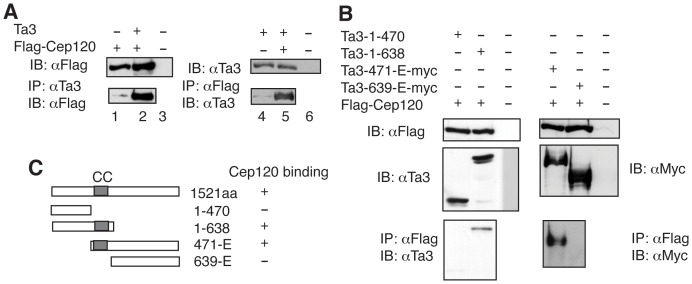
Ta3 interacts with Cep120 in the cell. FLAG-Cep120 was coexpressed with Ta3 (A) or various Ta3 mutants (B, C) in HEK293 cells as indicated. The protein lysates made from the cells were subjected to immunoblot (IB) or immunoprecipitation (IP) followed by immunoblot with the indicated antibodies. Note that the interaction of Cep120 with Ta3 requires the coil-coiled (CC) domain of Ta3 (B, C).

Ta3 is a centrosomal protein that is required for ciliogenesis and Hedgehog signaling [18], [19]. We used the yeast two-hybrid system to screen for Ta3-interacting proteins. One of the proteins identified using a Ta3 C-terminal bait was Cep120 (data not shown), another centrosomal protein [25]. The interaction between Ta3 and Cep120 was verified by coimmunoprecipitation using protein lysates prepared from HEK293 cells expressing both Ta3 and a Flag-tagged Cep120 (Fig. 1A). To map the region in Ta3 that is responsible for interaction with Cep120, coimmunoprecipitation was performed using four Ta3 expression constructs truncated from either the N- or C-terminus. The results showed that two truncated proteins, the 1–639 amino acid residues (aa) and 471-E aa fragments, were able to bind to Cep120, whereas the 1–470 aa and 639-E aa fratment were not (Fig. 1B, C). Thus, the region between 470 and 638 aa is essential for Cep120 binding.

**Figure 2:**
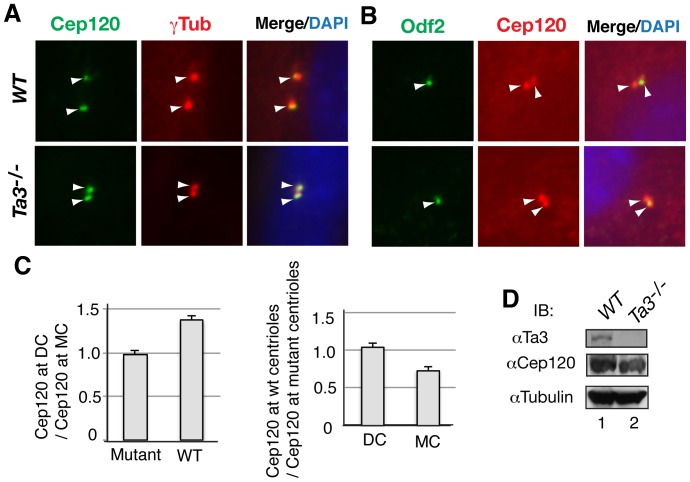
Ta3 is required for asymmetrical localization of Cep120 to the daughter centriole. Wild type and *Ta3* mutant primary mouse embryonic fibroblasts (pMEFs) were coimmunostained for Cep120 and γ ˜tubulin (A) or Odf2 and Cep120 (B), together with DAPI (for nuclei). Arrowheads indicate the specific staining at centrioles. The Cep120 signal at the daughter and mother centrioles (DC and MC, respectively) was quantified for 21 randomly chosen cells, using NIH image J. Cep120 signal ratios were then calculated, specifically the DC to MC ratio and the wild type to Ta3 mutant ratio (average + standard deviation) (C). (D) No significant changes in Cep120 levels in *Ta3* mutant MEFs. Immunoblots show the expression of endogenous Ta3 and Cep120 in wild type and *Ta3* mutant MEFs, with tubulin as a loading control.

To determine Cep120 subcellular localization, mouse embryonic fibroblasts (MEFs) were immunostained for both Cep120 and γ ˜tubulin, a centriole marker. We found that the intensity of Cep120 staining at daughter centrioles was consistently higher than that at mother centrioles (Fig. 2A, upper panels), thus confirming a recent study [20].

The molecular basis of asymmetrical Cep120 localization is not known. To determine whether Ta3 regulates Cep120 subcellular localization, we generated *Ta3* mutant MEFs, which lack cilia. Immunostaining of the MEFs for Cep120 now showed similar Cep120 signal intensity among the mother and daughter centrioles (Fig. 2, lower panels), indicating that Ta3 is required for the asymmetrical localization of Cep120 to daughter centrioles.

The loss of the asymmetrical centriolar localization in *Ta3* mutant cells could be due to either a decrease in Cep120 levels at daughter centrioles or an increase in its amount at mother centrioles. To distinguish between these two possibilities, both wild type and *Ta3* mutant cells were coimmunostained for both Cep120 and Odf2, an appendage marker of the mother centrioles [26] (Fig. 2B). Quantification analysis of Cep120 staining showed that the average ratio of Cep120 levels at daughter centrioles to those at mother centrioles was about 1 in the mutant cells, versus 1.4 in wild type cells (Fig. 2C, left panel). This again confirmed the even distribution of Cep120 to daughter and mother centrioles in *Ta3* mutant cells. Interestingly, Cep120 levels at daughter centrioles were about the same in mutant and wild type cells (about 1∶1 ratio), whereas at the mother centrioles, they significantly increased in mutant cells relative to wild type cells (1∶0.74 ratio) (Fig. 2C, right panel). This increase in Cep120 protein levels at mother centrioles of *Ta3* mutant cells was not due to an increase in expression, since total Cep120 expression levels in the mutant cells were comparable to those in wild type cells (Fig. 2D). Thus, Ta3 is necessary for limiting Cep120 localization at the mother centrioles in wild type cells.

### Loss of Cep120 results in hydrocephalus and cerebellar hypoplasia

**Figure 3:**
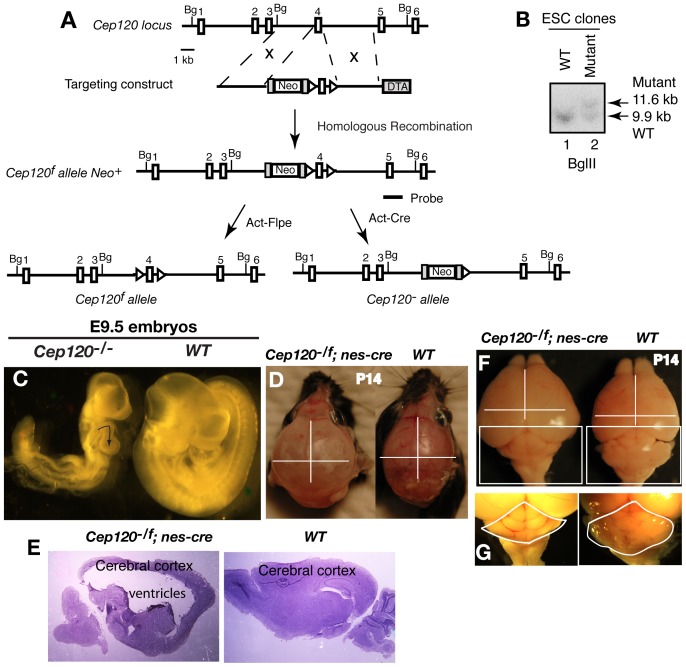
Loss of Cep120 results in early embryonic lethality, hydrocephalus, and cerebellar hypoplasia in mice. (A) The gene targeting strategy used to create mouse *Cep120^-^* and *Cep120^f^* mutant alleles. Open rectangles refer to exons (which are numbered), lines to introns, grey rectangles to Frt sites, and triangles to loxP sites. BglII (Bg) restriction sites and a probe for Southern blot are indicated. Neo, neomycin gene; DTA, diphtheria toxin A gene. (B) Southern blot analysis shows a representative mutant and wild type (wt) ES cell clones. (C) Lateral view of wild type and *Cep120^-/-^* embryos. Note that the development of Cep120 mutant embryos is delayed and the heart loops in the opposite direction (indicated by the arrow). (D–G) Morphology of two-week-old (P14) unskinned mouse heads (D), brains (E, F), and cerebellums (G), with indicated genotypes. The lines are in the same length for both wt and mutant, thus indicating the relative brain size or the extent of hydrocephalus in the mutant. Hematoxylin and eosin (H&E) staining of sagittal brain sections (E) confirms that the mutant ventricles of the brain are severely dilated. The boxed area in F is enlarged in (G). Cerebellums are outlined. The mutant cerebellum is significantly smaller (F).

To investigate the *in vivo* function of Cep120, a targeted gene knockout approach was taken to generate a mouse Cep120 conditional mutant allele named *Cep120^f^* (f refers to flox). This allele contains two loxP sites flanking exon 4 of the *Cep120* gene. The deletion of this exon is expected to result in a reading frame shift so that no functional protein would be produced. To generate a Cep120 null mutant allele, *Cep120^-^*, the exon was deleted by breeding *Cep120^f/+^* mice with *actin-Cre* transgenic mice (Fig. 3A, B). *Cep120^-/-^* mice died around gestation day 9.5, but with earlier developmental arrest at E8.5. Compared to wild type embryos, the mutant embryos failed to bend, and heart looping occurred in the opposite orientation (Fig. 3C).

To determine the role of Cep120 in the development of the central nervous system (CNS), *Cep120^+/-^* mice were crossed with *nes-Cre* mice to generate *Cep120^+/-^*; *nes-Cre* males, which were subsequently bred with *Cep120^+/f^* or *Cep120^f/f^* females to produce *Cep120^f/-^*; *nes-Cre* or *Cep120^+/+^*, *nes-Cre* mice. *nes-Cre* is expressed in the CNS including the developing cerebellum [27], [28]. *Cep120^f/-^*; *nes-Cre* newborns did not display any noticeable abnormalities. However, they began to develop hydrocephalus by postnatal day 7, or P7, which worsened the following week (Fig. 3D–E). The mice also lost motor balance and coordination and failed to grow (data not shown). Since the cerebellum controls motor movement and balance, both wild type and mutant brains at P14 were dissected and examined for morphologic differences. The mutant cerebral cortices were noticeably larger than those of wild type due to the accumulation of cerebrospinal fluid (CSF) (Fig. 3E–F). Additionally, the cerebellum failed to develop and lacked its typical foliation pattern (Fig. 3F–G). Taken together, these results indicate that Cep120 is essential for embryonic CNS development.

### Cep120 is required for the proliferation of cerebellar granule neuron progenitors (CGNPs) and cerebellar foliation

**Figure 4:**
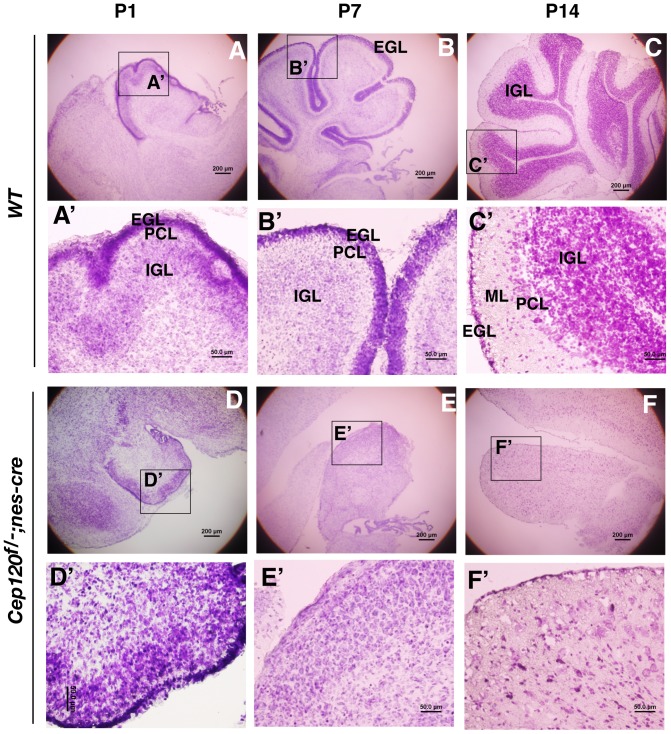
The Cep120 mutation results in cerebellar hypoplasia. Sagittal sections of P1, P7, and P14 wild type and *Cep120^f/-^*; *nes-Cre* mutant cerebellums were stained with cresyl violet. Framed areas are enlarged in the corresponding lower panels. EGL, external granule cell layer; PCL, Purkinje cell layer; ML, molecular layer; and IGL, inner granule layer.

CGNPs localized in the cerebellar EGL undergo massive proliferation after birth. These cells then exit the cell cycle and migrate into the cerebellar cortex to establish the IGL underneath the Purkinje cell layer (PCL). Associated with CGNP proliferation, differentiation, and migration is cerebellar foliation [3]. To determine which step of cerebellar developmental processes might be affected by the loss of Cep120 function, the cerebellar sections of P1, P7, and P14 wild type and mutant mice were subjected to cresyl violet staining to visualize GNPs. The results showed that the P1 mutant cerebellum had a very thin EGL compared to that of the wild type (Fig. 4, compare A and A″ to D and D″). GNPs in P7 and P14 wild type cerebellum underwent significant proliferation, differentiation, and migration to establish the IGL (Fig. 4, compare B and C to A). However, only a small number of GNPs were detected in the P7 and P14 mutant cerebellum. The mutant cerebellum showed developmental arrest at birth and a complete lack of foliation (Fig. 4E–F). The results suggest that Cep120 is essential for CGNP proliferation and cerebellar foliation.

**Figure 5:**
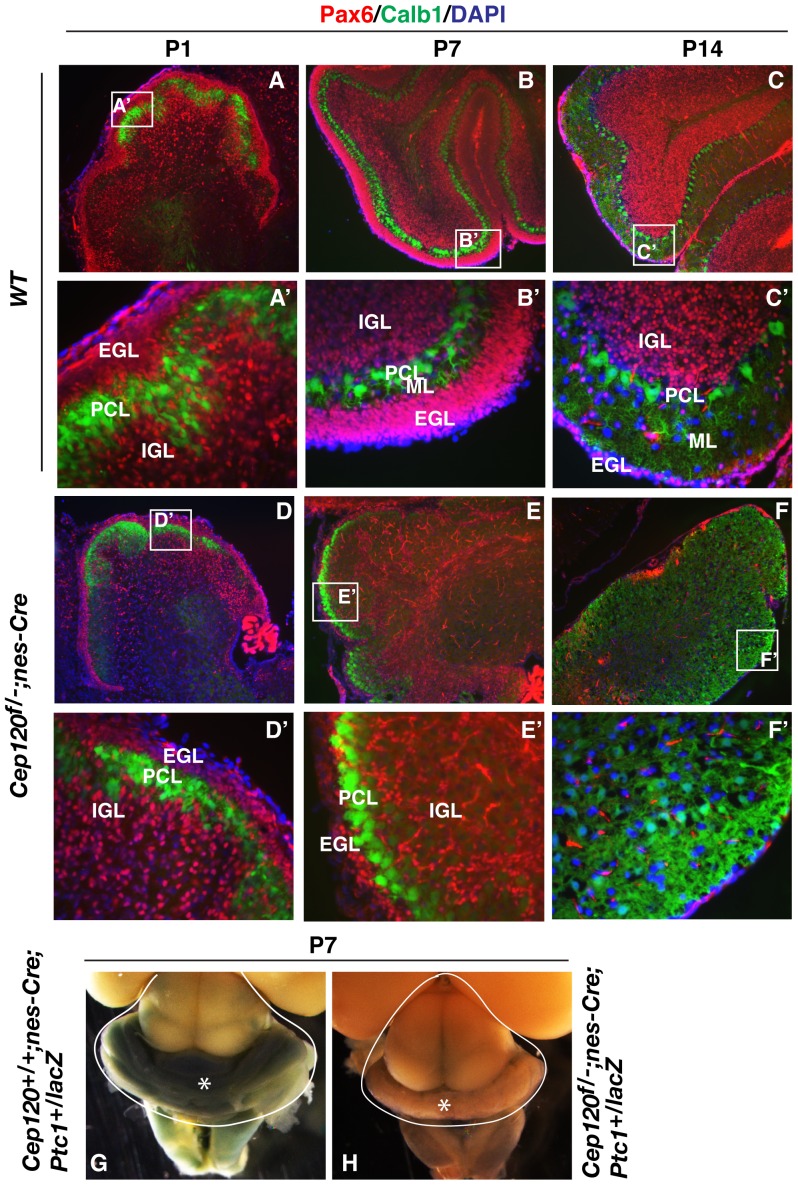
Loss of Cep120 results in failed expansion of granule neuron progenitors (GNPs), due to lack of a response to Hedgehog signaling. (A–F) Sagittal sections of P1, P7, and P14 wild type and mutant cerebellums were coimmunostained for Pax6 (red), Calb1 (green), and nuclei (DAPI, blue). Pax6 and Calb1 label GNPs and Purkinje cells, respectively. Framed areas in panels A–F are enlarged in A′–F′. EGL, external granule cell layer; PCL, Purkinje cell layer; ML, molecular layer; IGL, inner granule layer. Genotypes are shown to the left. (G–H) LacZ staining of the P7 cerebellum is positive in wild type animals (G), but negative in the Cep120 mutant (H). Genotypes are indicated on both sides. Cerebellums are outlined, and cerebellar posterior lobes are indicated by asterisks.

CGNP proliferation is normally induced by Shh secreted from the PCL underlying the EGL in the developing cerebellum [6]. The lack of CGNP proliferation could be due to either the absence of a PCL, an inability of the CGNPs to respond to Shh signals, or something entirely independent of Shh signaling. To rule out the first possibility and confirm the cresyl violet staining results, both wild type and *Cep120^f/-^*; *nes-Cre* cerebellums at P1, P17, and P14 were coimmunostained for Pax6 and Calb1 (Calbindin 1), which label GNPs and PCL, respectively [29]. The results showed that the PCL was present in the mutant cerebellum. In contrast, GNP number in EGL and IGL is slightly lower in P1 mutant cerebellum and reduced even more at P7, and barely detectable at P14 (Fig. 5, compare A–C′ to D–F′). To distinguish between the two remaining possibilities (failure of GNPs to respond to Shh or a Shh signaling-independent defect), we generated *Cep120^+/+^*, *nes-Cre*; *Ptch1^+/lacZ^* and *Cep120^f/-^*, *nes-Cre*; *Ptch1^+/lacZ^* mice. *Ptch1* encodes the receptor for Hh and is also the direct Hh target gene. *Ptch1^lacZ^* is a *Ptch*1 mutant allele in which the *lacZ* gene has been inserted in the *Ptch1* locus and thus serves as a reporter for Hh signaling [30]. LacZ staining showed that at P7, lacZ was highly expressed in the *Cep120^+/+^*; *nes-Cre*; *Ptch1^+/lacZ^* cerebellum but not in the *Cep120^f/-^*, *nes-Cre*; *Ptch1^+/lacZ^* mutant cerebellum (Fig. 5, compare G to H). Since residual GNPs, which normally express Ptch1, were generated in the P7 mutant cerebellum (Fig. 5E, E′), these observations indicate that the lack of CGNPs in p14 *Cep120* mutant mice (Fig. 5F, F′) most likely results from their failure to respond to Shh signals. This in turn leads to failed CGNP proliferation and differentiation.

**Figure 6:**
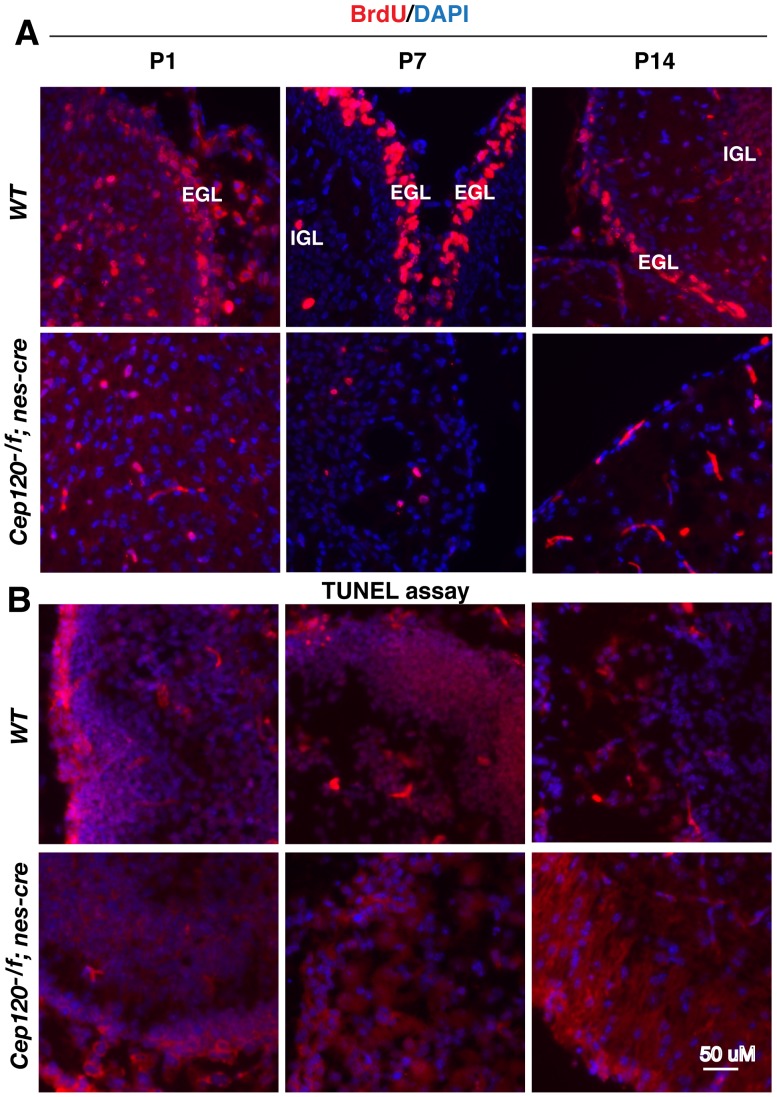
Failed proliferation of granule neuron progenitors (GNPs) in the Cep120 mutant cerebellum. (A) BrdU labeling showed BrdU incorporated into a significant number of GNPs in the wild type cerebellum (upper panels), but not in the Cep120 mutant (lower panels). (B) A TUNEL assay showed that no significant number of cells in Cep120 mutant cerebellum had undergone apoptosis. Representative areas of the cerebellum are shown. Nuclei are stained with DAPI (blue). EGL, external granule layer; and IGL, inner granule layer.

The cerebellar hypoplasia seen in Cep120 mutants could be caused by either failed GNP proliferation, increased GNP apoptosis, or both. In wild type cerebellums, GNPs proliferate very rapidly as indicated by the large fraction of GNPs being labeled with BrdU, particularly in the EGL. However, only a small number of GNPs were BrdU positive in the P1 *Cep120^f/-^*;*nes-Cre* mutant cerebellum, and the number was even fewer in P7 and P14 mutant cerebellums (Fig. 6A, compare wild type to mutant). In contrast, no significant cell apoptosis was observed in both wild type and mutant CGNPs as shown by TUNEL assay (Fig. 6B). Taken together, these findings indicate that the cerebellar hypoplasia in Cep120 mutants is due to a reduced GNP proliferation but not apoptosis.

### Cep120 is required for centriole duplication, maturation, and ciliogenesis

**Figure 7:**
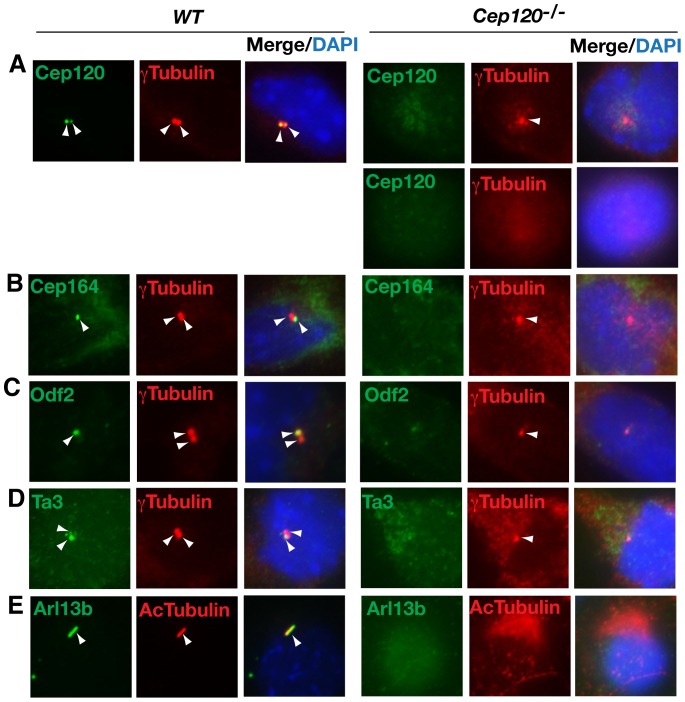
Cep120 is required for centriole duplication and ciliogenesis. Wild type and *Cep120^-/-^* pMEFs were coimmunostained for the indicated proteins. Most Cep120 mutant cells lacked either one or both centrioles, as indicated by γ ˜tubulin staining. Staining for Cep164, Ta3, and Odf2 showed that the missing centriole was the mother centriole. Staining for Arl13b and acetylated α ˜tubulin indicated no cilia being formed in Cep120 mutant cells. Arrowheads point to the staining of γ ˜tubulin (a centriole marker), the indicated proteins, or cilia.

To determine the cellular mechanism underlying the Cep120 mutant phenotype, we asked whether centriole duplication was affected, as Cep120 is a centrosomal protein [25]. The vast majority of wild type pMEFs each contained two centrioles, as indicated by positive immunostaining for γ ˜tubulin. However, most of the Cep120 mutant pMEFs lacked either one or both centrioles (Fig. 7A, compare wild type to mutant). To determine whether the remaining single centriole was a daughter or mother centriole, the cells were immunostained for Cep164 and Odf2, two appendage markers for mother centrioles [26], [31]. In wild type cells, both proteins were detected right at the mother centrioles. However, no staining was detected for either protein in the Cep120 mutant cells (Fig. 7B–C). The cells were also immunostained for Ta3, which was previously reported to localize equally to mother and daughter centrioles [19]. However, we found that in wild type cells, Ta3 predominantly localized to mother centrioles. This discrepancy could be due to a difference in the antibodies used recognizing different epitopes. Meanwhile, the stronger staining signal was absent in the mutant cells (Fig. 7D). Thus, the remaining single centriole in Cep120 mutant cells was the daughter centrioles. Additionally, since cilia originate in the mother centrioles, one would expect that Cep120 mutant cells would also lack cilia. Indeed, that was the case as evidenced by the absence of staining for acetylated tubulin and Arl13b, two cilia markers (Fig. 7E). These results indicate that Cep120 is required for centriole duplication, maturation, and subsequently ciliogenesis.

**Figure 8:**
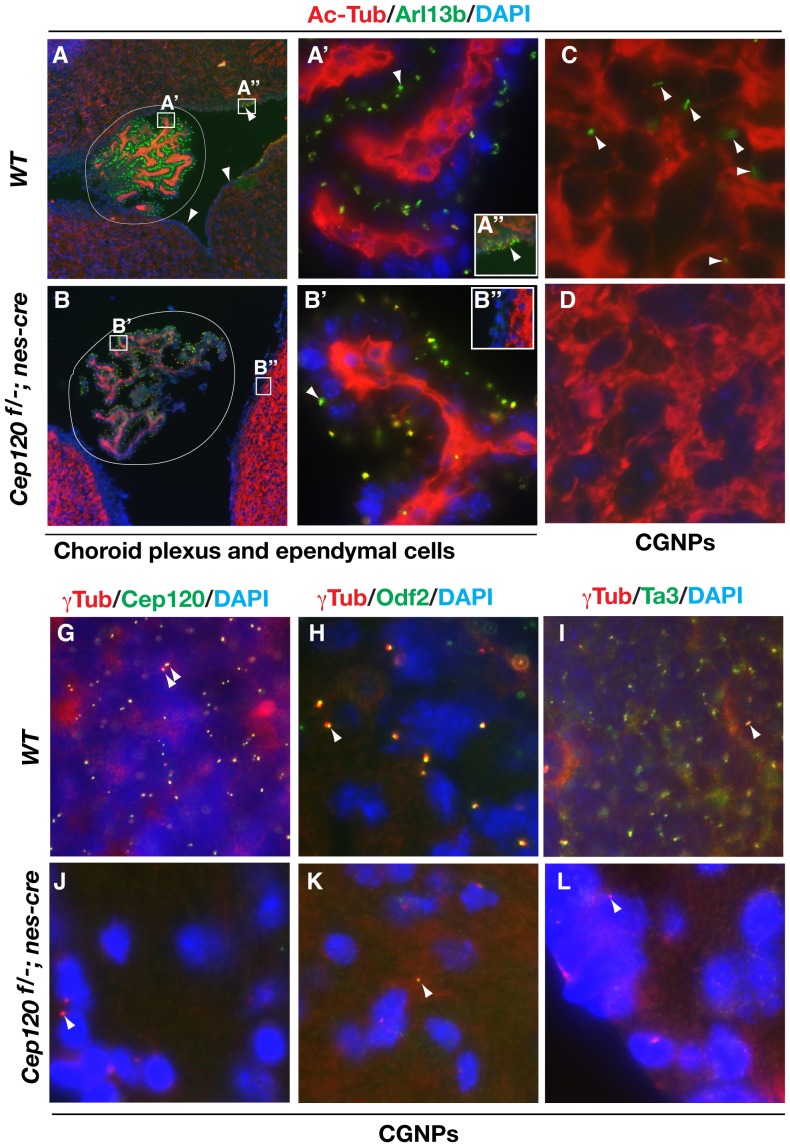
Failed centriole duplication, maturation, and ciliogenesis in cerebellar granule neuron progenitors (CGNPs) and ependymal cells in the Cep120 mutant. (A–D) Sagittal brain sections of P14 mice with the indicated genotypes were coimmunostained for acetylated α ˜tubulin and Arl13b, cilia markers. The fourth ventricular choroid plexus is circled. Framed areas in panels A and B are enlarged in A′/A″ and B′/B″. Panels A″ and B″ show representative areas of ependyma near the fourth ventricle. Panels C and D show representative areas of CGNPs. Arrowheads indicate representative cilia. Note that cilia develop in the *Cep120^f/-^*; *nes-Cre* choroid plexus, but not in ependymal cells and CGNPs. (G–L) Sagittal cerebellar sections of P14 mice with the indicated genotypes were coimmunostained for γ ˜tubulin and Cep120, Odf2, or Ta3, as shown. Note that very few centrioles are present in *Cep120^f/-^*; *nes-Cre* CGNPs, relative to wild type CGNPs. Arrowheads indicate one or two representative centrioles in each panel.

Cerebrospinal fluid (CSF) is produced largely by the choroid plexus of the brain. The CSF circulates through the ventricles of the brain, from the lateral ventricles to the third ventricle, through the cerebral aqueduct into the fourth ventricle, and finally into the spinal canal and subcarachnoid space. From there, it is absorbed into the venous and lymphatic systems [32]. Normal CSF flow requires the coordinated beating of cilia on the ependymal cells that line the ventricles. Ciliary dysfunction in either the choroid plexus or the ependymal cells has been shown to lead to hydrocephalus [32], [33]. To investigate whether the hydrocephalus of Cep120 mutants was caused by defects in cilia, sagittal brain sections were immunostained for both acetylated tubulin and Arl13b. Cilia were detected in choroid plexus in both the wild type and *Cep120^f/-^*; *nes-Cre* choroid plexus, though cilia numbers appeared slightly lower in the mutant. In contrast, although cilia were densely distributed on ependymal cells in the wild type brain, no cilia were detected on the mutant ependymal cells (Fig. 8A–B″, compare wild type to mutant). Thus, the development of hydrocephalus in the Cep120 mutant is most likely due to the loss of cilia on ependymal cells.

To explain the cerebellar hypoplasia seen in the Cep120 mutants, we also determined whether ciliogenesis and centriole duplication and maturation were impaired in the mutant CGNPs. Cilia were detected on some of the wild type CGNPs, but not on the *Cep120^f/-^*; *nes-Cre* CGNPs (Fig. 8, compare C to D). Similarly, although most wild type CGNPs each contained two centrioles, as determined by γ ˜tubulin- and Cep120-positive staining, most Cep120 mutant cells lacked both γ ˜tubulin and Cep120 staining. As in the mutant pMEFs, residual single centrioles in the Cep120 mutant CGNPs were daughter centrioles, based on negative Odf2 and Ta3 staining (Fig. 8, compare G–I to J–L). Therefore, the cerebellar hypoplasia of Cep120 mutants results from failed centriole duplication and maturation and ciliogenesis.

## Discussion

In the present study, we identified Cep120 as a Ta3-interacting protein. Cep120 localized asymmetrically to daughter centrioles, and this was dependent on Ta3. The *Cep120* mutation resulted in early embryonic lethality in mice. Conditional knockout of Cep120 in the CNS led to hydrocephalus and cerebellar hypoplasia. The latter was primarily attributed to a failure of GNP expansion. Cell biological studies revealed that Cep120 regulates centriole duplication and maturation, which is necessary for ciliogenesis. These findings for the first time linked a centrosomal protein required for centriole duplication to cerebellar and embryonic development.

The Ta3 C-terminal bait used for the yeast two-hybrid screen that identified Cep120 contained the 900-E amino acid residues. Paradoxically, coimmunoprecipitation results indicated that only the 471-E, and not the 639-E, fragment was able to interact with Cep120 even though both fragments contained the bait region. In addition, Cep120 could only interact with the Ta3-1-638, and not 1–471, fragment. This suggests that the 471–638 aa region is required for Cep120 binding (Fig. 1). This region contains the centrosomal localization domain [19]. Thus, one likely explanation for the discrepancy between the yeast two-hybrid and coimmunoprecipitation results is that the centrosomal localization of Ta3 is essential for its interaction with Cep120 in mammalian cells, whereas the localization is irrelevant in yeast, since the bait is artificially localized to the nucleus.

Ta3 is a centrosomal protein that localizes to both mother and the daughter centrioles and is required for ciliogenesis and Hh signaling [18], [19]. Our current study showed that more Ta3 localized to the mother centriole than the daughter centriole (Figs. 7D and 8I). However, Cep120 localized more to the daughter centriole than to the mother centriole (Fig. 2A) [20]. The molecular basis underlying this asymmetrical localization is unclear. In this study, we showed that Ta3 interacted with Cep120. This interaction was dependent on a middle region of Ta3 that was necessary for centrosomal localization (Fig. 1) [19]. Loss of Ta3 disrupted the asymmetrical localization of Cep120 to the daughter centriole. Quantification analysis revealed that this was due to an increase in Cep120 levels at mother centrioles, and not a decrease in its levels at daughter centrioles (Fig. 2B–C). Thus, Ta3 limits Cep120 levels at mother centrioles, which may be essential for mother centriole maturation. In this sense, Ta3 does not appear to regulate the actual Cep120 localization, rather probably serves as a sensor to control Cep120 levels at the mother centriole. We speculate that Ta3 may somehow facilitate the degradation of Cep120 specifically localized at the mother centriole by recruiting the protein degradation machinery to the proximity of the mother centriole.

Cells in G0 and G1 usually contain two centrioles. In ciliated cells, the mother centrioles mature and become the basal body of the primary cilium. During the cell cycle, cilia dissemble, and the existing centrioles replicate only once to produce two new centrioles. Thus, the two daughter cells each receive two centrioles at the end of the cell cycle, and their mother centrioles again form the base of the cilia [17]. Because centriole duplication and ciliogenesis are intricately linked, a perturbation of centriole duplication would also disrupt ciliogenesis. Recent RNAi knockdown studies showed that Cep120 was required for centriole duplication and elongation [20], [34], [35]. The present study provided genetic evidence for the role of Cep120 in centriole duplication. In addition, given that the single remaining centrioles in Cep120 mutant cells are the daughter centrioles, the result also suggests that Cep120 may be required for centriole maturation. Since cilia originate from the specialized mother centriole, the lack of cilia in Cep120 mutant cells was a consequence of failed centriole duplication, maturation, and the subsequent lack of the mother centriole, rather than ciliogenesis per se.

The left-right patterning of embryos is dependent on functional nodal cilia, and mutations in many known ciliary genes result in abnormal left-right embryonic patterning [36]. Since Cep120 mutant cells lack cilia, the aberrant heart looping of Cep120 mutant embryos was presumably due to the defect in nodal ciliogenesis. On the other hand, Cep120 mutant embryos died around E8.5, much earlier than many other known ciliary mutants. Therefore, the lethality most likely resulted from the loss of another Cep120 function, unrelated to cilia. Nevertheless, this function is likely related to mitotic spindle organization since centrosomes are responsible for this important process.

Defects in ciliogenesis have been shown to result in hydrocephalus. Two mechanisms are proposed to explain this. One is that a lack of motile cilia on ependymal cells results in impaired CSF flow and subsequent aqueductal stenosis [33]. The other is that loss of cilia leads to altered function of the choroid plexus epithelium [37]. In *Cep120^f/-^*; *nes-Cre* mutant mice, cilia were formed on epithelial cells in the choroid plexus, but not on ependymal cells (Fig. 8, compare As to Bs). Presumably Cre is not expressed in the choroid plexus. The result suggests that the lack of motile cilia on ependymal cells is the cause of hydrocephalus in the Cep120 mutant.

Studies have also shown that dysfunctional cilia are associated with cerebellar hypoplasia, and primarily attributable to a failure of GNP expansion [13], [14]. Nevertheless, a thin layer of EGL is still generated in IFT88 and Kif3a mutant cerebellums, and a residual IGL is also formed, indicating that some GNPs are still able to proliferate to expand the GNP pool. Associated with this is the partial foliation found in the mutant cerebellums. The results from BrdU incorporation and TUNEL assays indicate that cerebellar hypoplasia of IFT88 and Kif3a mutants is primarily due to reduced GNP proliferation. Although the Cep120 mutation also leads to cerebellar hypoplasia, the phenotypes are more severe than those of previously reported ciliary gene mutants. First, residual GNPs are detected only in P1 and P7, but not P14, mutant cerebellums, indicating that the GNPs barely proliferate after P7. Second, the Cep120 mutant cerebellum completely lacks any foliation (Figs. 4, 5A–F′). Thus, the cause of the cerebellar hypoplasia of Cep120 mutant appears more than just ciliary dysfunction. It could be due to the loss of another Cep120 function, unrelated to cilia, and more likely related to mitotic spindle assembly and positioning. This is normally regulated by centrosomes and is essential for the expansion and maintenance of neural progenitors during brain development [38].

CGNP proliferation is normally induced by Shh signals from Purkinje cells, and CGNPs fail to expand in the absence of Shh signaling [5], [6]. In the Cep120 mutant cerebellum, Shh is presumably expressed, since the Purkinje cell layer does form (Fig. 5D and E). Yet the lacZ inserted at the *Ptch1* locus is not expressed (Fig. 5H), although residual CGNPs are generated. Thus, the failed CGNP expansion is likely due to an inability to respond to Shh signaling, not decreased cell proliferation in the Cep120 mutant cerebellum. Since defects in cilia often lead to a similar failure to respond to Hh signaling [9], the lack of cilia in the Cep120 mutant is likely the mechanism underlying the absent Shh response.
